# Protein Attachment Mechanism for Improved Functionalization of Affinity Monolith Chromatography (AMC)

**DOI:** 10.3390/molecules27144496

**Published:** 2022-07-14

**Authors:** Nayan Nayak, Rosalinda Mazzei, Lidietta Giorno, João G. Crespo, Carla A. M. Portugal, Teresa Poerio

**Affiliations:** 1 LAQV-REQUIMTE, Department of Chemistry, NOVA School of Science and Technology, FCT NOVA, Universidade NOVA de Lisboa, 2829-516 Caparica, Portugal; nayaknayan@gmail.com (N.N.); jgc@fct.unl.pt (J.G.C.); 2 Institute on Membrane Technology, National Research Council, ITM-CNR, Via P. Bucci, 17/C, I87030 Rende, Italy; l.giorno@itm.cnr.it (L.G.); t.poerio@itm.cnr.it (T.P.)

**Keywords:** monoliths, APTES functionalization, chromatography, protein-binding kinetics, protein G, immunoglobulin G purification, protein immobilization

## Abstract

This work aims at understanding the attachment mechanisms and stability of proteins on a chromatography medium to develop more efficient functionalization methodologies, which can be exploited in affinity chromatography. In particular, the study was focused on the understanding of the attachment mechanisms of bovine serum albumin (BSA), used as a ligand model, and protein G on novel amine-modified alumina monoliths as a stationary phase. Protein G was used to develop a column for antibody purification. The results showed that, at lower protein concentrations (i.e., 0.5 to 1.0 mg·mL^−1^), protein attachment follows a 1st-order kinetics compatible with the presence of covalent binding between the monolith and the protein. At higher protein concentrations (i.e., up to 10 mg·mL^−1^), the data preferably fit a 2nd-order kinetics. Such a change reflects a different mechanism in the protein attachment which, at higher concentrations, seems to be governed by physical adsorption resulting in a multilayered protein formation, due to the presence of ligand aggregates. The threshold condition for the prevalence of physical adsorption of BSA was found at a concentration higher than 1.0 mg·mL^−1^. Based on this result, protein concentrations of 0.7 and 1.0 mg·mL^−1^ were used for the functionalization of monoliths with protein G, allowing a maximum attachment of 1.43 mg of protein G/g of monolith. This column was then used for IgG binding–elution experiments, which resulted in an antibody attachment of 73.5% and, subsequently, elution of 86%, in acidic conditions. This proved the potential of the amine-functionalized monoliths for application in affinity chromatography.

## 1. Introduction

In affinity chromatography, immobilized binding agents are seen to be used with various supports. In the past, particulate-based materials such as silica particles, agarose beads, glass beads, membranes, and particulate supports prepared from several organic polymers have been utilized for this purpose [1,2,3,4,5,6]. Nevertheless, the scientific community has witnessed an increasing interest in incorporating affinity chromatography with monolithic supports [5,6,7,8,9,10,11]. This blend has recently been referred to as affinity monolith chromatography, or AMC [9,10,11]. Modern monoliths, developed as supports for affinity chromatography, seem to offer several advantages [5,6,7,8,9,10,11]—including better efficiency, lower backpressure requirements, and increased permeability—in comparison to particle-based supports. These characteristics are beneficial, specifically for affinity-based separations to operate at higher fluxes, such as rapid flow-based immunoassays. Furthermore, high efficiency is required for certain applications, such as high-throughput drug screening and chiral separations [8,9,10,11].

Copolymers of ethylene dimethacrylate (EDMA) and glycidyl methacrylate (GMA) are presently the most commonly available monoliths [12,13,14,15,16,17,18,19,20,21,22]. These monoliths are known to have a facile method of synthesis, and can be modified to have a hydrophilic form with traits of lower nonspecific binding for several biological agents. However, their main disadvantage as a support is that they tend to exhibit a smaller surface area while comparing them to the traditional particulate supports. Thus, the amount of binding agent to immobilize on a support is limited [8,9,10,11,23,24,25]. Other alternatives, such as agarose, offer several advantages, including low nonspecific binding for many biological matrices, higher stability for a broad pH range, ease of modification to immobilize several binding agents, and the intrinsic property of larger pore size. Their major drawback for use in applications involving high-performance liquid chromatography (HPLC) is that they are fragile and display lower mechanical stability [1,5]. In the present work, the affinity monolith chromatography (AMC) potential of previously fabricated and functionalized amine-modified alumina monolithic columns was analyzed [26,27]. A significant amount of literature is available about the surface properties and interaction behavior of proteins with monolith supports [28,29,30], but not so much about protein attachment mechanisms in immunoaffinity-based chromatography. In addition, since the interactions are mainly related to the type of support and surface properties, the kind of immobilization strategy, and the biomolecule properties (tendency to aggregate, etc.), a deeper knowledge about protein attachment mechanisms is essential in order to optimize the proteins involved (e.g., antibodies, protein G) and, consequently, partially reduce the high process costs associated with the need for such expensive ligands. In this regard, extensive protein binding studies were carried out, using bovine serum albumin (BSA) as a model protein, to understand the interaction between the column and protein, and how it influences the binding capacity of the amine-functionalized monoliths. The attachment was characterized in terms of kinetics, and the mechanism of binding was speculated so as to understand how a globular protein such as BSA or protein G might interact with the monolithic column. The binding stability was tested under stressing conditions, such as acidic and basic pH, high ionic strength, and high temperature. Subsequently, attachment with protein G using two different concentrations was tested. Finally, these protein G columns were used to assess the binding and elution of immunoglobulin G as a model system.

## 2. Results

The main goal of this work was to understand the influence of protein concentration on the efficiency of the monolithic column functionalization with specific ligands, such as protein G. The aim was to optimize protein-binding methodologies, which may lead to affinity chromatography systems with the highest loads of effective and stably bound ligand (protein G), with minimal ligand shortages and wastage.

### 2.1. Understanding Protein Attachment Mechanisms with APTES-Functionalized Alumina Monoliths

**Figure 1:**
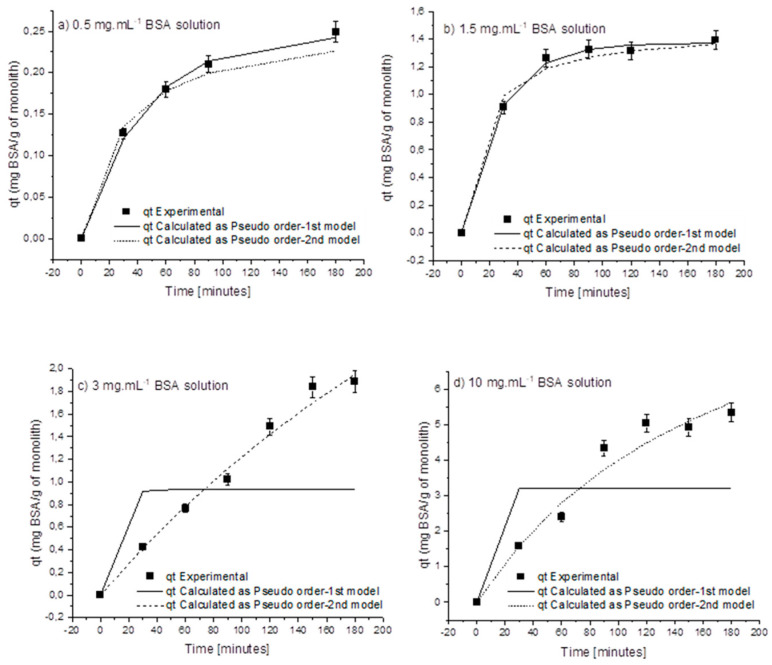
Fitting of qt (mg BSA/g of monolith) data as a function of time with pseudo-1st-order and pseudo-2nd-order kinetic models, using initial BSA concentrations: (**a**) 0.5 mg·mL^−1^ BSA, (**b**) 1.5 mg·mL^−1^ BSA, (**c**) 3 mg·mL^−1^ BSA, and (**d**) 10 mg·mL^−1^ BSA.

Initial assays were performed using bovine serum albumin (BSA, pI = 4.7, molecular weight = 66.5 kDa) as a model protein, and then assays with the target ligand (protein G) were carried out at a later stage of these studies. Despite the differences in the molecular sizes of BSA (~67 kDa) and protein G (22–34 kDa), their globular shape and similar isoelectric point (IEP) (IEP_BSA_ = 4.8 [31], IEP_Protein G_ = 3.7–4.4) [32] justified the selection of BSA to mimic the interactions of protein G with the amine-functionalized monolith. There are many reported mechanisms for the attachment of BSA to the amine groups [33]. The best possible conditions would need to be provided for the binding to proceed favorably. In the present case, the ideal binding conditions were ensured by using protein solutions at pH 7, mimicking the physiological conditions. This pH value lies between the isoelectric points (IEPs) of BSA (pH ~4.8) and APTES (pH ~11.4), thus guaranteeing that the amine groups on the monolith remained protonated (positively charged) and BSA had a negative net charge. Note that APTES was initially bound to the monolith column, as reported in Section 3, to be used as a source of amine groups needed for protein attachment. Experiments were carried out at varying BSA concentrations, from 0.5 mg·mL^−1^ to 10 mg·mL^−1^. The protein solution was recirculated through the column, and samples were taken at fixed intervals up to a period of 3 h. The kinetic profiles of BSA’s reaction with the amine-functionalized alumina monolith are shown in Figure 1. These data were fitted to two different rate equations, corresponding to a pseudo-first-order model and a pseudo-second-order model. The parameters of the fitting and the obtained constants are shown in the table below (Table 1).

The data indicate changes in BSA attachment profiles for different initial concentrations of protein (from 0.5 to 10 mg·mL^−1^). The obtained results show that at lower concentrations (0.5–1.5 mg·mL^−1^), the data prevalently fit to pseudo-first-order kinetic models, but as the protein concentration increases to values higher than 1.5 mg·mL^−1^, the protein attachment profiles shift clearly to pseudo-second-order kinetics. Pseudo-second-order kinetic models are typically used to describe adsorption phenomena; therefore, it appears that as the concentration is increased, the attachment switches to adsorption.

**Figure 2:**
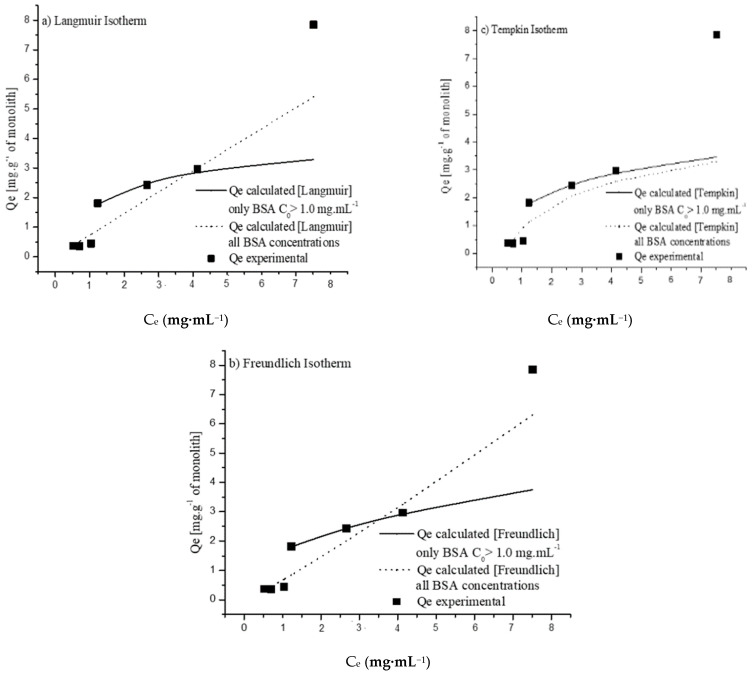
Fitting of BSA adsorption data at the equilibrium (Q_e_ (mg·g^−1^ of monolith)) obtained as function of concentration remaining in solution at the adsorption equilibrium (Ce), varying BSA initial concentration (0.5–5 mg/mL) with (**a**) Langmuir, (**b**) Freundlich, and (**c**) Tempkin isotherms.

To better understand the attachment of proteins to the monoliths, the BSA adsorption isotherms were determined (mass of protein adsorbed per mass of monolith at the equilibrium, Q_e_ vs. the protein concentration remaining in solution at the adsorption equilibrium, C_e_) and fitted to the most commonly used adsorption models, namely, Langmuir, Freundlich, and Tempkin models. As can be seen in Figure 2, the data obtained for concentrations below 1.5 mg·mL^−1^ do not fit adequately to any of the isotherms tested, suggesting that the dominant process in this concentration range is not adsorption. However, as the concentration increases above 1.5 mg·mL^−1^, it appears that the data fit into certain adsorption isotherms, as shown in Figure 2. The data values obtained for initial protein concentrations ranging from 1.5 mg·mL^−1^ to 5 mg·mL^−1^ (inclusively) fit acceptably to all of the three of the models tested, suggesting that protein adsorption might be the dominant protein attachment mechanism in this concentration range. However, it seems that a different trend might exist at a concentration of 10 mg·mL^−1^, indicating an evolution of protein attachment to a different mechanism, possibly related to the presence of protein aggregation phenomena, which might be enhanced at increasing protein concentrations. The effects of protein concentration and fluid flow are discussed later in this paper.

**Figure 3:**
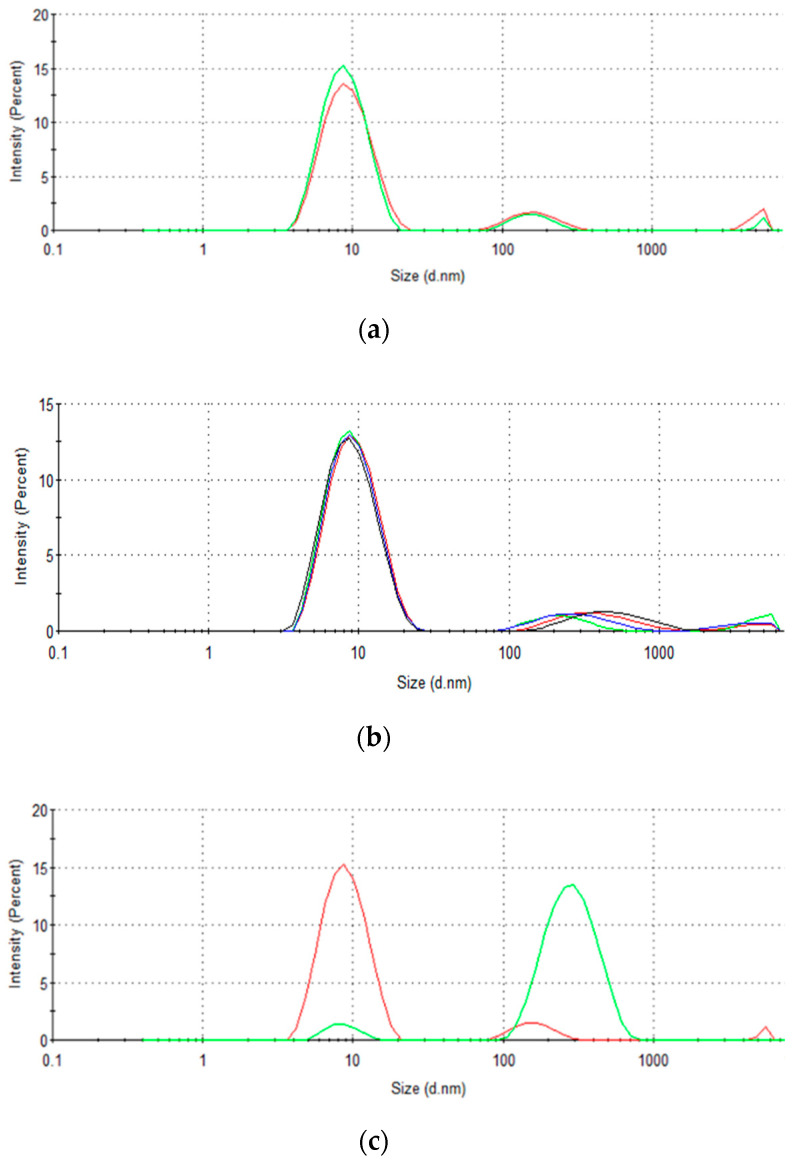

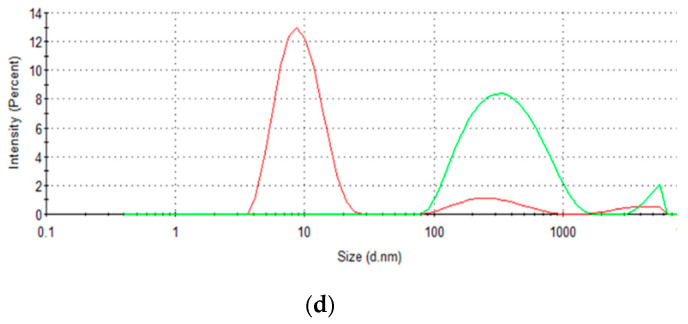
Effect of the protein concentration on the particle size distribution in (**a**) 0.5 mg·mL^−1^ (red line) and 1.0 mg·mL^−1^ (green line) BSA solutions, and (**b**) 1.5 mg·mL^−1^ (red line), 3 mg·mL^−1^ (blue line), 5 mg·mL^−1^ (green line), and 10 mg·mL^−1^ (black line) BSA solutions without recirculation through the amine-functionalized monolith column. Effect of contact time of the protein solution with the monolith on the particle size distribution when using (**c**) 1 mg·mL^−1^ and (**d**) 5 mg·mL^−1^ BSA solutions in static conditions (red line) and recirculated through the monolith column for 24 h (green line).

The values of the maximum adsorption amount (Q_m_), correlation coefficient (R^2^), chi-squared (χ^2^), and the other parameters for all of the isotherms are shown in Table 2. The Langmuir isotherm [34] assumes that adsorption occurs on a homogeneous surface containing sites with equal energy and that are equally available for adsorption. This is valid for the complete monolayer of adsorption, on which there is no transmigration of the adsorbate on the surface plane. A Q_m_ value of of 3.95 mg·g^−1^ was obtained for this isotherm, with an R^2^ value of 0.97, showing acceptable fitting of this isotherm to the experimental data at higher BSA concentrations (Figure 3a.)

The Freundlich isotherm [35] is an empirical equation that can be used for heterogeneous systems, and in addition to protein–material interactions it also considers interactions between adjacent adsorbed molecules. The n_F_ parameter, known as the heterogeneity factor, can be used to indicate whether the adsorption trend is linear (n_F_ = 1) and whether it is a chemisorption (n_F_ < 1) or physisorption (n_F_ > 1) process [36]. The values of n_F_ = 2.46 and 1/n_F_ = 0.41 indicate that the physical process and cooperative multilayered attachment are favorable. The fitting of the Freundlich isotherm to the experimental data (R^2^ = 0.99) is shown in Figure 3b. Like the Freundlich isotherm, the Tempkin isotherm [37] considers the interactions between adsorbate molecules, assuming that the adsorption heat of all molecules decreases linearly after the layer that is initially adsorbed on the monolith surface is covered, and that the adsorption has a maximum energy distribution of uniform bond. The constant b_T_ is the Tempkin isotherm constant, and may be used to calculate the value of heat of adsorption, B = RT/b_T_, where R is the universal gas constant (R = 8.314 J·K^−1^·mol^−1^) and T is the temperature in Kelvin (K). A low value of heat of adsorption (B = 0.92 kJ/kmol) was obtained, indicating that the adsorption is mainly a physical phenomenon. The fit to experimental data (R^2^ = 0.98) in Figure 3c shows that the Tempkin isotherm is quite adequate to explain the adsorption of BSA onto the amine-modified monolith (as is the Freundlich isotherm).

Dynamic light scattering (DLS) measurements were performed to study changes in protein aggregation in solution, aiming to understand the different BSA binding/adsorption trends with respect to protein concentration as well as contact time with the monolith while recirculating (simulated stirring). For freshly prepared solutions, a bimodal distribution was observed for all concentrations (Figure 3a,b). A first band appeared invariably with the maximum centered at ~9 nm, in agreement with the molecular size of a protein monomer, whereas a second band with lower intensity appeared at larger molecular sizes (~300–600 nm). The increase in BSA concentration did not have a significant impact on the first band, which remained predominantly centered at ~9 nm. However, a slight shift of the maximum of the second band to higher particle sizes—more significant for a protein concentration of 10 mg·mL^−1^—was observed. This is indicative of a slight increase in BSA aggregation with the increase in protein concentration.

The influence of the elution conditions on particle size distribution was also analyzed. The results depicted in Figure 3c,d clearly show a shift in the population of particle sizes caused by the shear stress conditions associated with fluid recirculation. As shown in Figure 3, the dynamic contact with the monolith leads to an increase in the population of larger particles of 100–1000 nm (2nd band), to the detriment of the population of smaller particles (1st band). This behavior was even more significant in the case of solutions with higher BSA concentrations. The results obtained with the BSA solution at a concentration of 5 mg·mL^−1^ are shown in Figure 3d to illustrate the strong fluid-flow-induced aggregation effect at higher protein concentrations. In fact, at higher protein concentrations, the signal of monomers appears to be negligible with respect to the protein aggregates, indicating a more sluggish Brownian motion due to the presence of larger and heavier particles. Based on these observations, it could be hypothesized that the predicted “layer-upon-layer” attachment may only correspond to the attachment of large and heavy aggregates to free attachment sites in the monolith, at higher BSA concentrations.

It appears that the protein attachment to the column may be more complicated than expected. From the standpoint of applications, it is crucial to obtain stable protein–monolith interactions. Therefore, the attachment stability of the protein to the monolith columns was elicited under diverse conditions. As it was assumed that the electric charges of BSA and APTES, along with buffer pH, play key roles in the protein attachment to the column, it was imperative to determine whether BSA attachment was governed by electrostatic interactions. If this is the case, it should be possible to elute most of the protein by using buffers with pH < 4.8 (where both BSA and APTES are positively charged) or pH > 11 (where both BSA and APTES are negatively charged). Experiments to access protein elution were carried out for 19 h with 2 mL of glycine–HCL (pH = 3.1) and glycine–NaOH (pH = 11.7), with phosphate buffer (pH = 7.0) washes in between. The amount of protein dislodged was quantified by BCA tests, which revealed that a maximum of only 8–10% of the attached protein was detached from the column. Not considering the functionality, this result suggests that the protein attachment itself was very stable.

Subsequently, to check whether the protein was attached due to other weak interactions, such as hydrogen bonds, the column was also eluted with buffer solutions with ionic strengths of 5 mM and 0.1 M NaCl at a temperature of 50 °C. In this case, it was observed that less than 5% (by weight) of the attached protein was desorbed. Again, this showed that the protein was very stably attached to the column.

To confirm that the protein was stably bound to the column, the column was crushed, and the protein attached to the resultant powder was estimated through the BCA test. It was found that all of the undetached BSA was indeed present within the column. These results seem to confirm that the protein was stably attached to the column, or that the formation of protein aggregates during the protein attachment process may impair the release of the aggregates by elution due to size exclusion effects, resulting in the maintenance of protein in the column.

Considering these results with BSA, it is suggested that, in order to avoid aggregation and ensure that the column is optimally functionalized with ligands—in this case, protein—lower-concentration solutions (below 1 mg·mL^−1^) must be used in the functionalization process. This is ideal, as it will avoid wastage of the expensive protein G.

Subsequently, protein G adsorption studies were carried out with 0.7 and 1 mg·mL^−1^ solutions of protein G.

**Figure 4:**
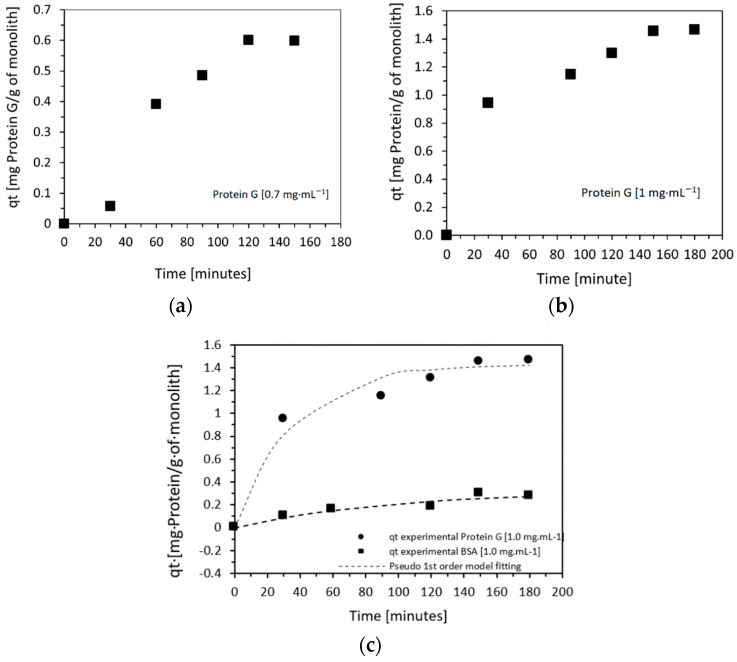
Amount of Protein G, qt (mg Protein G/g monolith) attached to the amine-functionalized monolith column as a function of time using initial Protein G concentrations of (**a**) 0.7 mg·mL^−1^ and (**b**) 1.0 mg·mL^−1^. (**c**) Amounts of protein G and BSA, qt (mg of protein/g of monolith) attached to the amine-functionalized monolith column as a function of time, using initial protein concentrations of 1 mg·mL^−1^.

Figure 4a,b show the kinetics of attachment of protein G. The experiment was conducted with initial protein G concentrations of 0.7 and 1.0 mg·mL^−1^, and the experiment was run for a total of 3 h. At the end of this process, the amount of protein G bound to the column was determined by BCA tests. Protein G attachment values of 0.61 mg of protein/g of monolith (0.91 mg of protein G/cm^3^ of monolith) in the case of an initial protein G solution of 0.7 mg·mL^−1^, and 1.43 mg of protein/g of monolith (2.1 mg protein G/cm^3^ of monolith) when 1.0 mg·mL^−1^, were used.

A significant increase in the amount of attached protein was observed with respect to BSA, as can be seen in Figure 4c, due to both the lower molecular weight of protein G and its lower tendency to aggregate [38,39,40]. The mass of protein G attached was sevenfold higher than that of BSA. However, considering the lower molecular weight of protein G, these mass differences translate into ~14× more protein G molecules attached to the column compared to the number of attached BSA molecules.

In the range of concentrations used, as previously observed for BSA, the data related to protein G immobilization also fit pseudo-first-order kinetics (pseudo-first-order R^2^ = 0.965; pseudo-second-order R^2^ = 0.834).

### 2.2. Performance of Protein-G-Functionalized Alumina Monoliths for IgG Binding and Elution

A binding experiment was carried out to test the capacity of the protein-G-functionalized amine–monolith column to bind immunoglobulin G (IgG). IgG at a concentration of 1 mg·mL^−1^ in 50 mM phosphate-buffered saline, at pH = 7.0, was recirculated in the column at a flow rate of 0.47 mL·min^−1^.

**Figure 5:**
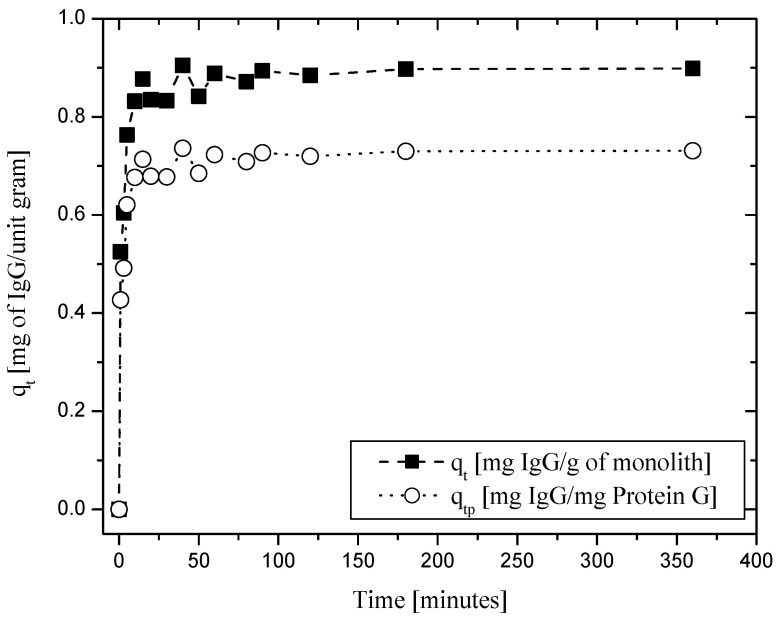
Capacity of the amine–monolith column functionalized with protein G to bind immunoglobulin G (IgG), expressed in terms of the mass of IgG adsorbed per mass of monolith and mass of protein G in the monolith.

As shown in Figure 5, 0.89 mg (5.93 × 10^−3^ mol) of IgG adsorbed per g of column (1.53 mg of IgG/cm^3^ of monolith or 1.02 × 10^−3^ mol IgG/cm^3^ of monolith) was readily captured, corresponding to 73.5% saturation of the protein G immobilized in the column (i.e., 0.731 mg IgG/mg of protein G or 4.87 × 10^−3^ mol of IgG/mg of protein G). Subsequently, when eluted using a 25 mM citrate buffer (pH = 3.5), about 0.89 mg of IgG was recovered after 5 h of elution, representing 86% of the total mass of IgG attached to the column (1.31 mg IgG/cm^3^ monolith). This value, as expected, was one order of magnitude smaller than typically found in conventional resin chromatographic columns [41], due to the lower specific surface area of monolith systems. However, it is noteworthy that although the elution was carried out for 5 h, the recovery of IgG took place in less than 50 min.

As discussed in a previous work [27], based on these results it is clear that the amine functional groups in the alumina column assure a stable attachment of BSA and protein G. However, protein aggregation might have a significant influence on the binding of BSA, depending on the concentration of the initial protein solution. Furthermore, based on the protein elution studies performed using buffer solutions at extreme pH values and varying salt concentrations, it appears that the protein interacts with the APTES via a strong bond, as suggested in previous studies [30]. Finally, binding and elution experiments using IgG showed that such a column can effectively be used for affinity-based purification of immunoglobulins.

## 3. Materials and Methods

### 3.1. Materials

α-Alumina (CT-3000) from Alcoa Chimie (Milano, Italy), Dolapix PC 67 from Zschimmer & Schwarz (Lahnstein, Germany), gelatin porcine skin (Oxoid-LP0008) and sodium lauryl sulfate/SDS (L-6026) from Sigma-Aldrich, and commercially available corn oil were used to prepare the alumina monoliths. The alumina monoliths were functionalized with (3-aminopropyl) tetraethoxysilane (APTES) supplied by Sigma-Aldrich. Heat-shrink Teflon tubes were purchased from an online store. Fittings for the columns were obtained from BioToolomics PLC, UK. Analytical grade disodium hydrogen phosphate and sodium dihydrogen phosphate were used to prepare a 50 mM phosphate buffer (PB) solution at pH = 7. A standard bicinchoninic acid (BCA) test (reagents sourced from Thermo Fisher, Monza, Italy) was used to measure the protein concentration. Bovine serum albumin (BSA) from Sigma-Aldrich (Milano, Italy) was used as model protein in the experiments. Glycine–HCl (pH = 3.1) and glycine–NaOH (pH = 11) buffers at concentrations of 0.1 M and 1 M, as well as 5 mM and 0.1 M NaCl solutions, were used for protein desorption. Recombinant protein G > 98% was purchased from BioVision. Human IgG was obtained from Sigma-Aldrich Chemical Co. (Milano, Italy).

### 3.2. Fabrication of α-Alumina Monoliths

The alumina monolithic columns were prepared as described in our previous papers [26,27]. Briefly, a 50% (*v*/*v*) alumina suspension was prepared in water. The mass of alumina needed to obtain this solution was calculated using the density of the reactive alumina powder, which was equal to 3.98 g·cm^−^^3^ [26]. Dolapix was added as the decoagulant. Emulsions were prepared by adding this suspension to corn oil (V_oil_/V_suspension_ = 1.5). Sodium dodecyl sulfate (SDS) and gelatin were also added to this mixture, acting as a surfactant and gelling agent, respectively. This mixture was stirred at 1000 rpm, and the resultant emulsions were poured into cylindrical molds and, once set, the obtained green bodies were sintered with a specific thermal cycle. The sintered columns were further activated by immersion in H_2_O_2_ (incubated at 90 °C for 120 min) for removal of organic content [27]. Subsequently, the obtained monolith was modified through the sol–gel method with (3-aminopropyl)tetraethoxysilane (APTES) as the silane agent, in ethanol, using NaOH as the catalyst, in order to obtain an amine-functionalized monolithic column. The process was carried out in an environment with 100% relative humidity at 80 °C for 150 min. Furthermore, the samples were dried for 16 h at 60 °C. These columns were then chosen for further studies of protein attachment.

### 3.3. Preparation of Affinity Monolith Chromatography Setup

**Figure 6:**
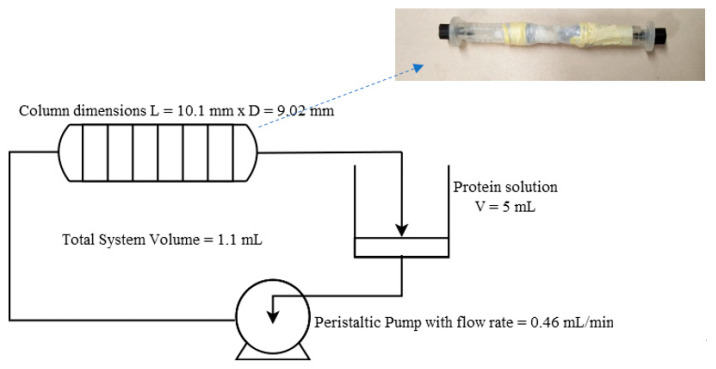
Schematic representation of process setup.

The amine-functionalized alumina monoliths were initially weighed (dry weight); then, they were inserted into Teflon tubing, and then a short heat treatment was applied externally to the tube using a standard Bunsen burner to shrink the tube so that it would tightly wrap around the monolith, making it watertight around the sides to avoid preferential draining through the walls. The HPLC fittings were then attached to the ends of the tube (Figure 6). The setup was assembled as shown in the schematic representation in Figure 6. The heat-shrink Teflon-tube-wrapped monoliths, modified with APTES, were connected to 5 mL reservoirs containing the protein solution, through a peristaltic pump. This setup configuration was used to ensure the complete wetting of the highly porous monoliths. Standard HPLC fittings were used for the connections. The pumps were calibrated before each run, and the final flow rates were also verified after each attachment cycle.

### 3.4. Interaction of Proteins with Amine-Functionalized Monoliths and Attachment Kinetics

Protein interaction with the amine-functionalized columns was tested initially using BSA as the model protein, and then using protein G, which is known to offer good binding affinity with antibodies. The column was initially equilibrated by recirculating 5 mL of 50 mM phosphate buffer (pH = 7.0) for 1 h. Next, the phosphate buffer solution was replaced with 5 mL of the protein solution (BSA or protein G), which was added to the reservoir. The pump was set to a flow rate of 0.47 mL·min^−1^. Time was recorded as soon as the column was filled. Samples (100 µL) were taken every 30 min for the first 3 h, for quantification of the protein attached to the column over time. Hence, a total sample volume of about 0.7 mL was removed from the 5 mL reservoir. The sampled volume was considered for the calculations. After 3 h, the process was stopped, and the column was emptied and rapidly washed with 5 mL of fresh buffer in single-pass mode. Subsequently, a recirculated washing with fresh buffer (PB) was carried out for a further 1.5 h to remove the unbound proteins. The first-stage experiments were carried out using BSA solutions with initial concentrations of 0.5, 1.0, 1.5, 3.0, 5.0, and 10.0 mg·mL^−1^. In the second stage, these experiments were repeated using protein G, with initial concentrations of 0.7 and 1 mg·mL^−1^. Each experiment was repeated at least three times.

The obtained protein binding data (mg of protein/g of column vs. time) were fitted to the pseudo-first-order and pseudo-second-order kinetic equations shown in Equations (1) and (2), respectively:

(1) $$q_{t}=q_{e}[1-\exp(-k_{1}t)]$$

(2) $$q_{t}=\frac{k_{2}q_{e}^{2}t}{1+k_{2}q_{e}t}$$

where *k*_1_ is the rate constant for the pseudo-first-order adsorption (min^−1^), *k*_2_ is the rate constant for the pseudo-second-order adsorption (g_monolith_/mg_protein_ min), and *q_e_* (mg of protein/g monolith) and *q_t_* (mg of protein/g monolith) correspond to the monolith adsorption capacity in equilibrium and at a given time “*t*”, respectively.

Additionally, the obtained protein adsorption data—i.e., protein concentration at the equilibrium vs. initial protein concentration—were fitted to the Langmuir, Freundlich, and Tempkin isotherms depicted in Equations (3)–(5), respectively:

(3) $$q_{e}=\frac{Q_{m}K_{a}C_{e}}{1+K_{a}C_{e}}$$

(4) qe=KFCe1nF

(5) $$q_{e}=\frac{RT}{b_{T}}\ln(k_{T}C_{e})$$

where *Q_m_* (mg protein/g monolith) is the maximum adsorption capacity, *C_e_* (mg·mL^−1^) is the equilibrium concentration of the solute (BSA or protein G), *K_a_* (mg·mL^−1^) is the Langmuir constant, *K_F_* (mL·g^−1^) and *n_F_* are the Freundlich constants, *b_T_* (J·moL^−1^)’ and *k_T_* (mL·mg^−1^) are the Tempkin constants, *R* corresponds to the universal gas constant, and *T* is the absolute solution temperature (in Kelvin).

### 3.5. Protein-Binding Stability

The stability of the interaction between the proteins and the amine-functionalized columns was elicited by analysis of protein desorption, following the methods described below.

#### 3.5.1. Desorption Using Glycine–HCl and Glycine–NaOH (0.1 M and 1 M)

Separate experiments were carried out using a glycine–HCl buffer at a pH of 3.1 and a glycine–NaOH buffer at a pH of 11.0, as described in [27]. Briefly, the buffer was taken in the reservoir, and the experiment was carried out at a flow rate of 0.47 mL·min^−1^ for 19 h. A sample (control) was taken at the beginning of the experiment, and a final sample was collected after 19 h. As the pH values of these buffers were at extremes (3.1 and 11.0), the samples were initially treated with acetone to induce protein precipitation (as recommended by Thermo Fisher) [42]. The protein precipitate was then redissolved in phosphate buffer, at pH 7.0, for protein quantification through the BCA test.

#### 3.5.2. Desorption Using NaCl (5 mM and 0.1 M) and Temperature

Saline solution (2 mL) with varying ionic strengths (5 mM and 0.1 M) was taken in the reservoir, and the experiment was carried out at a flow rate of 0.47 mL·min^−1^ for 19 h. A sample (control) was taken at the beginning of the experiment, and a final sample was collected after 19 h. In addition, experiments were conducted at a temperature of 50 °C for each concentration. Protein quantification was performed using the BCA test.

#### 3.5.3. Quantification of the Total Protein Attached or Entrapped in the Monolith

To evaluate the total protein entrapped in the column after performing all of the stability tests, the column was crushed to a fine powder using a mortar and pestle. The protein content in this ground powder was quantified using the BCA test, using 0.1 g powder samples. The same amount of pure alumina powder and a powdered amine-functionalized alumina without attached protein were used as blank and control samples, respectively.

### 3.6. Inspection of Protein Aggregation Effects

The presence of potential protein aggregation effects was inspected by dynamic light scattering (DLS) analysis and reported as particle size distribution and polydispersity (PDI). The ZetaSizer 7.1 software provided with the instrument was used to collect and analyze the data. The samples were measured at a controlled temperature of 25 °C. DLS was used to measure the diffusion of particles subjected to Brownian motion, and by using the Stokes–Einstein relationship, the motion of particles was converted to its size and size distribution. This incorporated a noninvasive backscattering angle of 173°, and allowed the detection of particles in the range of 0.6–6000 nm. All studies were repeated thrice, and the values of the z-average diameters were used.

### 3.7. Evaluation of the Efficiency of Protein-G-Bound Amine-Functionalized Monolith for Purification of Immunoglobulin G (IgG)

The effectiveness of the amine-functionalized columns bound with protein G, to be used as an affinity chromatography system for the purification of immunoglobulin G (IgG), was analyzed. Initially, the column was equilibrated by recirculating 5 mL of phosphate buffer (pH = 7.0) for 1 h. To study the adsorption of IgG, 5 mL IgG solutions at 0.7 mg/mL and 1 mg/mL, in phosphate buffer (pH 7.0), were taken in the reservoir in two independent experiments. The pump was set to a flow rate of 0.47 mL·min^−1^. Time was recorded as soon as the column was filled. Samples (100 µL) were taken every 30 min for 3 h. Thus, a total sample volume of about 0.6 mL was used for analysis from the 5 mL reservoir. This volume change was compensated accordingly in the calculations. After 3 h, the process was stopped, and the column was emptied and rapidly washed with 5 mL of fresh buffer, without recirculation, to remove the unbound proteins. Subsequently, a recirculated wash with fresh buffer (PB) was carried out for a further 1.5 h to equilibrate the column. The elution of IgG was performed using a 25 mM citrate buffer at pH 3.5 under the same conditions as described above.

## 4. Conclusions

The interaction between BSA and the amine-functionalized column was studied in terms of attachment mechanism. Data fitting indicated that at lower protein concentrations a mechanism based on covalent attachment of BSA to the column was mainly present. However, as the concentration of BSA in the solution increased, there was a trend of protein aggregation, resulting in the appearance of multilayered attachment of the protein to the column—as indicated by fitting the data to the Freundlich isotherm. It was also observed that at lower BSA concentrations the binding followed a first-order kinetic model, while at concentrations higher than 1.5 mg·mL^−1^, the behavior switched to a second-order kinetic model. The presence of protein aggregation at higher protein concentrations was corroborated by dynamic light scattering studies, which confirmed the effects of higher protein concentrations and the recirculation through the monolithic column in promoting protein aggregation. Additionally, chemical stability to extreme-pH and -ionic-strength elution solutions indicated that BSA was very stably bound to the column at all concentrations.

Results related to BSA were used to design the optimal conditions for attachment of the more expensive protein G in order to avoid wastage of this protein, which is commonly used as a ligand for IgG purification by affinity chromatography. It was found that up to 1.43 mg of protein G/g of monolith was successfully bound to the column (at a solution concentration of 1.0 mg·mL^−1^). A subsequent trial with IgG adsorption on this column revealed that about 73.5% of IgG/protein G was bound to the column, and about 86% of this could be successfully eluted using an acidic buffer. This result indicates that the column has a good potential to be used in affinity chromatography.

## Figures and Tables

**Table 1:** Experimentally determined and theoretically predicted parameters for adsorption kinetics models.

Initial Concentration (mg·mL^−1^)	Q_Expt_ (mg·g^−1^)	Pseudo-First-Order				Pseudo-Second-Order			
		Q_Pred_ (mg·g^−1^)	k_1_	R^2^	χ^2^	Q_Pred_	k_2_	R^2^	χ^2^
						(mg·g^−1^)			
0.5	0.249	0.242	0.022	0.990	0.001	0.261	0.137	0.989	0.003
1	0.276	0.319	0.011	0.925	0.022	0.461	0.0175	0.928	0.020
1.5	1.395	1.374	0.037	0.997	0.003	1.474	0.0466	0.989	0.016
3	1.887	0.938	0.783	0.425	1.803	1.951	0.0003	0.988	0.025
5	2.239	0.678	0.783	0.275	3.729	2.037	0.0004	0.905	0.434
10	5.333	3.208	0.783	0.521	4.332	5.619	0.0005	0.964	0.229

**Table 2:** Isotherm parameters determined through fitting of adsorption data for BSA solutions with concentrations varying from 1.5 mg·mL^−1^ to 5 mg·mL^−1^.

Langmuir		Freundlich		Tempkin	
Q_m_	3.947068	K_F_	0.861167	K_t_	1.138941
K_a_	0.133524	n_F_	2.463419	b_T_	2735.478
χ^2^	0.006007	χ^2^	0.000692	B	0.920915
R^2^	0.97742	R^2^	0.997444	χ^2^	0.003577
				R^2^	0.986508

## Data Availability

The data are not publicly available since some of them can be integrated with other data and published.

## Sample Availability

Samples of the compounds are not available from the authors.
